# Longitudinal familial analysis of blood pressure involving parametric (co)variance functions

**DOI:** 10.1186/1471-2156-4-S1-S87

**Published:** 2003-12-31

**Authors:** Julia MP Soler, John Blangero

**Affiliations:** 1 Department of Statistics, University of Sao Paulo, Sao Paulo SP, Brazil; 2 Department of Genetics, Southwest Foundation for Biomedical Research, San Antonio Texas USA

## Abstract

**Background:**

For analyzing longitudinal familial data we adopted a log-linear form to incorporate heterogeneity in genetic variance components over the time, and additionally a serial correlation term in the genetic effects at different levels of ages. Due to the availability of multiple measures on the same individual, we permitted environmental correlations that may change across time.

**Results:**

Systolic blood pressure from family members from the first and second cohort was used in the current analysis. Measures of subjects receiving hypertension treatment were set as censored values and they were corrected. An initial check of the variance and covariance functions proposed for analyzing longitudinal familial data, using empirical semi-variogram plots, indicated that the observed trait dispersion pattern follows the assumptions adopted.

**Conclusion:**

The corrections for censored phenotypes based on ordinary linear models may be an appropriate simple model to correct the data, ensuring that the original variability in the data was retained. In addition, empirical semi-variogram plots are useful for diagnosis of the (co)variance model adopted.

## Background

Longitudinal designs in family studies represent additional opportunities to model temporal variation in genetic and environmental factors influencing quantitative traits. For certain phenotypes, like anthropometric measures and blood pressure, more insight into its continuous physiological variation may be provided for adopting phenotype (co)variance components as a function of time, rather than scalar components. In this article the term (co)variance is been used to refer both to variance and covariance components.

The classical approach to the genetic analysis of longitudinal traits, under the variance component framework, considers an unstructured covariance matrix [1] for modeling the correlations on the sequence of measurements within an individual. Without making assumptions about the (co)variance, terms the model is not flexible for incomplete profiles, and it is not clear how to define the heritability measures to incorporate the longitudinal features. One approach is to use structured (co)variance patterns, assuming, for instance, that the different variance components change across time with ages, according to a parametric function, and allowing the autocorrelation process within the measurements of the individuals. In this regard, gains in precision are obtained by reducing the number of parameters involved in the analysis.

Using the real longitudinal Framingham Heart Study data, we analyzed the systolic blood pressure as the trait of interest. To pursue the major genes influencing the trait, under the mixed-model longitudinal approach, we employed the genetic variance function  in the familial model, where *t *represents the age in the application. Under this parameterization, a possible heterogeneity of the genetic variance, in continuous time, is being considered through the parameter γ, which represents an interaction effect between genetic and environmental factors. If there is evidence of change in the genetic variance across time, the parameter γ is significantly different from 0. Additionally, the correlation in genetic effects at different ages (for instance, *t *and *s*) was modeled as ρ _*g *_= exp (- λ|*t *- *s*|), where λ is assumed different from 0.

To absorb the dependence in the sequential measurements within an individual, environmental correlations were added in the model. Data from family members from the first and second cohort that were re-examined 21 and 5 times, respectively, during the longitudinal phase of the study, were used in the current analysis. The precision of the estimates obtained from these analyses was compared. For subjects receiving hypertension treatment, the recorded systolic blood pressure was considered as censored value, and to accommodate it in the analysis, we proceeded with corrections using a nonparametric algorithm to adjust the censored phenotypes, as considered by Levy et al. [2]. Only systolic blood pressure measures taken when subjects were aged 18 years or more were supposed to be informative for the analysis.

## Methods

### Adjusted right-censored phenotypes

For subjects receiving hypertension treatment, the recorded systolic blood pressure was considered as a right-censored value, since one knows that it is less than what the untreated value would be. To accommodate the censoring process in the analysis we addressed corrections on the censored phenotypes through the nonparametric algorithm used by Levy et al. [2]. Separate adjustments were conducted according to sex and age groups (<35, 35 to 44, 45 to 54, 55 to 64, 65 to 74, and 75 years). In this phase of the analysis, for adjustment of the censored measurements, ordinary linear regression models were adopted to investigate the relationship between age and systolic blood pressure, despite multiple observations being available in the same individuals. Because assumptions about phenotype (co)variance structure can affect the fitted values, it is useful to have simple methods for correction of the censored responses. The residuals obtained were ordered to generate a sample from a discrete reference distribution. Censored phenotypes were adjusted by conditional expectations of the untreated residuals given residual values of equal or greater magnitude. Once untreated observations constituted the vast majority of the data, this replacement process should not reduce the original variability of the data [3].

### Longitudinal familial variance component model

To define the adopted model, let *Y*_*j*_(*t*) denote a measurement on the *j*^*th *^individual at the time *t*. The cross-sectional familial variance component approach [4] can be extended for longitudinal data. The polygenic model was defined as:



where μ is the overall mean, *X*_*jt *_is a covariates vector (describing, for instance, sex scores, linear and quadratic polynomial coefficients associated with levels of age, and possible interactions) at occasion *t *for the *j*^*th *^individual, β is a parameters vector associated with the fixed effects, *g*_*jt *_and *e*_*jt *_are uncorrelated random effects due to polygenic and environmental sources of variation, respectively. For relatives *i *and *j*, observed at *t *and *s *occasions, respectively, we assume:



where 2φ_*ij *_is the kinship coefficient between relatives *i *and *j*.

Following Aitkin [5], who considered a log-linear form for modeling the heterogeneity of the variance, we employed the polygenic variance function , where *t *denotes age. The parameter γ accommodates longitudinal changes in the trait heritability, and represents the interaction effect between the polygene and age. Since polygenic correlations between the expressions of the trait at different ages usually depends on their time range, we adopted a monotone decreasing function ρ(*t*,*s*)=*exp*(-λ|*t*-*s*|), as proposed by Diggle [6] and Huggins et al. [7]. Stating that ρ(*t*,*s*) is different from 1 suggests that different polygenes influence the trait in different ages. Also, modeling the correlation structure in continuous time admits unbalanced sequences of measurements on the different individuals.

To complete the specification of the model, because multiple measurements are available in the same individuals, we need to adopt parametric functions to the environmental components,  and σ_*e *_*(t, s)*. As stated before, log-linear variance function and serial correlation structure were assumed. Additionally, such parametric (co)variance functions may be extended for longitudinal familial oligogenic model. In this case, for major genes are attributed random effects, which are associated with familial covariance matrices structured in terms of identity-by-descent matrices.

To estimate the parameters involved in the specification of the (co)variance components under multivariate normal distribution and involving longitudinal and familial dependence patterns, we used a version of the EM (expectation maximization) algorithm, as described by Iturria and Blangero [8] and implemented into the software SOLAR. Hypothesis tests concerning the (co)variance parameters involved were conducted through likelihood ratio statistics.

### Empirical semi-variogram

The empirical semi-variogram of a sequence of measurements within the same individual is a scatter plot of squared differences of residuals,  against the corresponding lag of times (|*t *- *s*|). In this regard, the residuals are obtained from ordinary linear regression adjustment of the phenotype in terms of covariates involved in the study. We used the empirical semi-variogram to provide a valuable, but informal, initial check on the longitudinal (co)variance structure, as used by other authors [6,9].

## Results

During the analysis of the second cohort, we considered longitudinal measurements from 1667 individuals (822 male and 845 female), totaling 7177 observations. A vast majority of individual profiles (81%) contain four or five measurements throughout the study. The results of the adjustments for censored phenotypes receiving hypertension treatment are presented in Table 1. The greatest proportions of corrections occur for measurements with negative residuals, within males, and higher ages.

**Figure 1:**
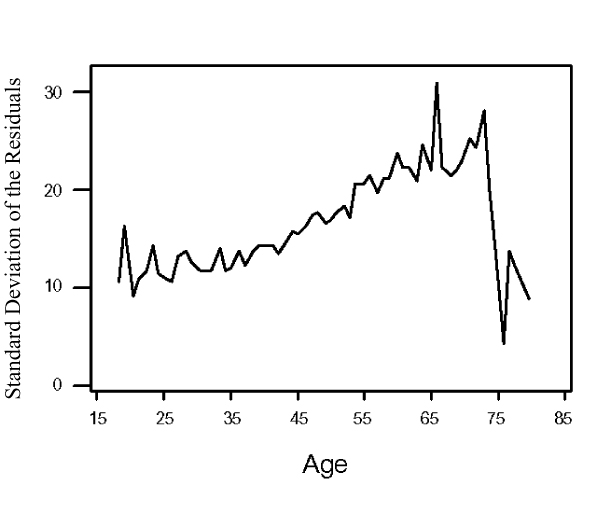
Residual variability according to age

**Figure 2:**
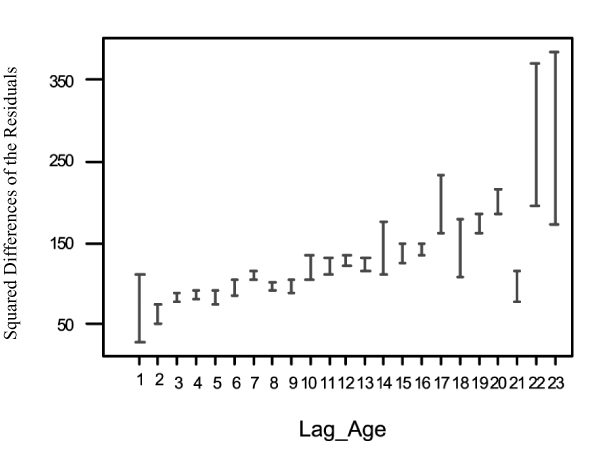
Empirical semi-variogram for real data set from second cohort.

Figure 1 shows standard deviation of ordinary least-square residuals plotted against ages. The plot suggests that the variances for the phenotype change with age. The log-linear dispersion pattern can represent an appropriate model, and will be adopted in the analysis. Additionally, Figure 2 plots empirical semi-variogram values as a function of the lag of ages. The non-random pattern shown implies that the contribution of the autocorrelation process is not negligible and needs to be absorbed in the analysis.

## Conclusions

In this work, we presented a methodology to analyze longitudinal familial data set, considering the adoption of parametric functions for modeling genetic and environmental (co)variance components involved. The approach is applied for trials of systolic blood pressure measurements. Considering that the hypertension treatment generates a right-censored response, we proceeded with corrections on the values observed under treatment. The corrections were based on information from residuals obtained through ordinary linear models, an appropriate simple model useful to estimation in this phase of the analysis. Because the vast majority of the data are untreated measures, such replacement ensures the original variability of the data, which will be modeled in terms of genetic and environmental (co)variance components. An initial check of the proposed parametric (co)variance functions, using empirical semi-variogram plots, indicated that the systolic blood pressure dispersion pattern follows assumptions as heterogeneity of variance and autocorrelation process. Having done these preliminary analyses, we are now involved in the estimation and hypothesis tests related to the genetic parameters of the proposed model.

## Figures and Tables

**Table 1:** Trait average to untreated (SBP), hypertension treated (SBP_Hy) and adjusted (SBP*_Hy) values

Age	N	# Hy	Residual < 0			Residual > 0		
			SBP	SBP_Hy	SBP*_Hy	SBP	SBP_Hy	SBP*_Hy
Male								
18–34	841	10	110.58	114.33	127.08	130.10	134.86	143.36
35–44	1082	50	112.90	107.90	126.35	132.82	135.68	145.76
45–54	926	104	116.20	117.30	135.40	138.52	143.31	158.77
55–64	517	134	120.51	123.67	148.88	148.22	151.20	168.79
65–74	139	55	123.67	128.83	161.73	155.00	158.19	181.08
75	12	6	124.33	127.25	148.96	153.00	156.00	157.56
Total	3517	359						
Female								
18–34	804	3	101.5	-	-	160.68	121.00	129.31
35–44	1080	49	103.26	108.60	123.17	124.34	134.10	145.37
45–54	1008	110	109.20	114.09	133.57	134.84	138.77	152.60
55–64	565	132	115.54	121.09	147.68	145.25	148.93	168.15
65–74	196	64	122.04	127.43	154.64	150.16	152.30	173.01
75	7	2	126.50	-	-	165.00	316.00	330.47
Total	3660	360						
